# CAH3 from *Chlamydomonas reinhardtii*: Unique Carbonic Anhydrase of the Thylakoid Lumen

**DOI:** 10.3390/cells13020109

**Published:** 2024-01-05

**Authors:** Vasily V. Terentyev, Anna K. Shukshina

**Affiliations:** Institute of Basic Biological Problems, FRC PSCBR RAS, 142290 Pushchino, Russia

**Keywords:** *Chlamydomonas*, CAH3, carbonic anhydrase, photosystem II, carbon-concentrating mechanism

## Abstract

CAH3 is the only carbonic anhydrase (CA) present in the thylakoid lumen of the green algae *Chlamydomonas reinhardtii*. The monomer of the enzyme has a molecular weight of ~29.5 kDa with high CA activity. Through its dehydration activity, CAH3 can be involved either in the carbon-concentrating mechanism supplying CO_2_ for RuBisCO in the pyrenoid or in supporting the maximal photosynthetic activity of photosystem II (PSII) by accelerating the removal of protons from the active center of the water-oxidizing complex. Both proposed roles are considered in this review, together with a description of the enzymatic parameters of native and recombinant CAH3, the crystal structure of the protein, and the possible use of lumenal CA as a tool for increasing biomass production in higher plants. The identified involvement of lumenal CAH3 in the function of PSII is still unique among green algae and higher plants and can be used to understand the mechanism(s) of the functional interconnection between PSII and the proposed CA(s) of the thylakoid lumen in other organisms.

## 1. Introduction

Spontaneous conversion between different forms of inorganic carbon (C_i_) occurs easily in water solutions. However, the reactions are relatively slow. The rate constants for CO_2_ hydration and HCO_3_^−^ dehydration at 25 °C are 0.030–0.037 s^−1^ and 24–26 s^−1^, respectively [1,2,3]. Widespread metal-containing enzymes in nature, carbonic anhydrases (CAs, EC 4.2.1.1), catalyze these reactions, i.e., the reversible hydration of CO_2_ (CO_2_ + H_2_O ↔ HCO_3_^−^ + H^+^), and human CAII, as the most active CA identified to date, may accelerate the reaction by factor 10^6^ [4].

Many CAs are found to participate in photosynthetic processes in cyanobacteria, algae, and higher plants (see reviews [5,6,7,8]), where they are mainly involved in the rapid conversion of CO_2_ to HCO_3_^−^ in the carbon-concentrating mechanism (CCM) or, vice versa, of HCO_3_^−^ to CO_2_ to be used by ribulose-1,5-bisphosphate carboxylase/oxygenase (RuBisCO, EC 4.1.1.39).

The need for CA activity near the donor side of photosystem II (PSII) has been proposed for a long time, since the participation of HCO_3_^−^ (bicarbonate) ions in the operation of the water-oxidizing complex (WOC) of PSII was assumed [9], and since a rapid release of H^+^ into the thylakoid lumen during the reaction of photosynthetic water oxidation was observed [10]. In addition, the influence of CA inhibitors, as well as the knockout of genes encoding some CAs in higher plants, have changed the activity of PSII, the state of PSII photoprotection mechanisms, and even PSII protein composition [11], despite the fact that the identification of the exact protein(s) in the close vicinity of PSII that have CA activity is still under investigation.

In the green algae *Chlamydomonas reinhardtii*, thirteen CAs of different families (α, β, γ) have been found to date [6,12], while the limiting CO_2_-inducible B protein (LCIB) of the chloroplast stroma, which does not have CA activity, was assigned to CAs of the β-family only recently [13]. Many CAs (together with C_i_ (inorganic carbon) transporters) are involved in the CCM [7,14], whose activity allows for increasing the intracellular pool of C_i_ (mostly in the form of HCO_3_^−^) up to 40–100-fold that of the CO_2_ level of the aquatic environment [7], to saturate RuBisCO for carboxylation [15]. The expression levels of genes encoding these CAs usually increase significantly in response to CO_2_ limitation, i.e., CCM activation. For example, the genes encoding CAH1, which is the major periplasmic CA in *C. reinhardtii*, and mitochondrial CAH4 and CAH5 are strongly up-regulated by low CO_2_ [16]. The expression level of the gene encoding LCIB is also up-regulated by low CO_2_ (the same has been observed for the gene encoding LCIC) [17]. However, the genes encoding CAH2 and CAH6 are not up-regulated by low CO_2_ [16] in spite of the fact that these two CAs are also classified as CCM participants [7,14]. The expression level of the gene encoding CAH2 is even decreased over one hour after the shift of algal cells to low CO_2_ [16]. 

Little is known about CAH7, CAH8, and CAH9. The expression levels of the genes encoding CAH7 and CAH8 are not regulated by low CO_2_ [16], while the gene encoding CAH9 is expressed at a low level [18]. Three mitochondrial γ-CAs (CAG1–3) in *C. reinhardtii* are probably part of mitochondrial complex I. However, there are no complete data about their roles [6]. 

A recent study about the localization of CCM components in the cell of *C. reinhardtii*, with use constructions encoding proteins of interest with a fluorescence tag, clarified the location of CAH2 (plasma membrane and late-secretory pathway), CAH4, CAH5, CAG1–3 (mitochondria), and CAH9 (cytosol) [19]. In addition, this study corrected the localization of β-CA CAH6 found in the flagella, where CAH6, as a CCM participant, can be involved in C_i_ sensing [19]. 

CAH3 is the third α-CA (after CAH1 and CAH2) found in *C. reinhardtii* and is the only CA whose location (and function) is associated with the thylakoid membrane of the algal chloroplast [20,21]. Initially, CAH3 located in the thylakoid lumen was also attributed to participants of the CCM [20], while a high amount of CAH3 protein was found in PSII-enriched membranes, which made it possible to suggest a functional interconnection between CAH3 and PSII [22,23]. A possible dual role of CAH3 in the thylakoid lumen of *C. reinhardtii*, i.e., participation in CCM and involvement in PSII function, was schematically represented previously [24]. 

Interestingly, to date, CAH3 from *C. reinhardtii* has been the only CA clearly observed in PSII preparations [10,23,25,26,27] isolated from cyanobacteria, algae, and higher plants whose activity supports the function of the WOC. This makes CAH3 a unique protein for the study of the involvement of CA(s) in the functioning of the photosynthetic apparatus in general and of PSII in particular. 

This review summarizes current knowledge about the structural and functional properties of native and recombinant CAH3 proteins, the proposed roles for CAH3 in photosynthetic processes of the algal chloroplast, and attempts to incorporate CAH3 into the chloroplasts of higher plants in order to increase their bioproductivity.

## 2. Chloroplast-Located CA Activity

In the early 1990s, the presence of two major periplasmic α-CAs in *C. reinhardtii* was well known: low-CO_2_-inducible CAH1 and high-CO_2_-inducible CAH2, which had tetrameric structures consisting of two large and two small subunits linked by disulfide bonds [28,29]. The sequence homology was high, ~92% [29], making it possible to assume that they were the result of gene duplication [28]. At the same time, the presence of chloroplast-associated CA activity depending on the CO_2_ level in the growth medium was detected (Table 1).

The use of a CA-directed photoaffinity reagent (^125^I-labeled *p*-aminomethyl-benzenesulfonamide-4-azidosalicylamid) allowed for the detection of a 37 kDa polypeptide in cell lysates associated with a large subunit of periplasmic CA(s), while a 30 kDa photoaffinity-labeled peptide localized predominantly in the membrane fraction was also observed, indicating the presence of intracellular CA [36]. The 30 kDa photoaffinity-labeled peptide was not detected in cell lysates from the *cia3* mutant of *C. reinhardtii* [36], in chloroplasts of which CA activity was almost completely suppressed (Table 1) [32,33] (see also p. 3).

Karlsson and coworkers [21] were able to isolate membrane-associated intracellular CA from the wall-less mutant CC-503 cw92 (mt+) (CC-503), which had a high CA activity of 1260 WAU mg^−1^ associated with a single polypeptide at around 29.8 kDa. The protein did not show cross-reaction with the antibody against periplasmic CA from *C. reinhardtii* or pea chloroplastic CA. A 50% inhibition of CA activity was observed with 12 nM acetazolamide (AZA). Based on the sequence of the N-termimi, which has a part identical to *C. reinhardtii* Fd-NADP^+^, the target of the precursor should be the chloroplast [21].

It was later shown that thylakoid membranes isolated from CC-503 have ~15 times higher CA activity than thylakoid membranes from the *cia3* mutant at the same chlorophyll (Chl) content, while the presence of EZA strongly decreased CA activity in the case of preparations from CC-503 in contrast to preparations isolated from *cia3* [23]. The CA activity of membranes enriched by PSII was additionally ~3.4 times higher than that observed for thylakoids from CC-503 [23]. At the same time, the adaptation of CC-503 cells to low-CO_2_ conditions led to a ~5.5-fold increase in CA activity associated with thylakoids [37].

## 3. Amino Acid Sequence of the CAH3 Protein and Immuno-Analysis

**Figure 1:**
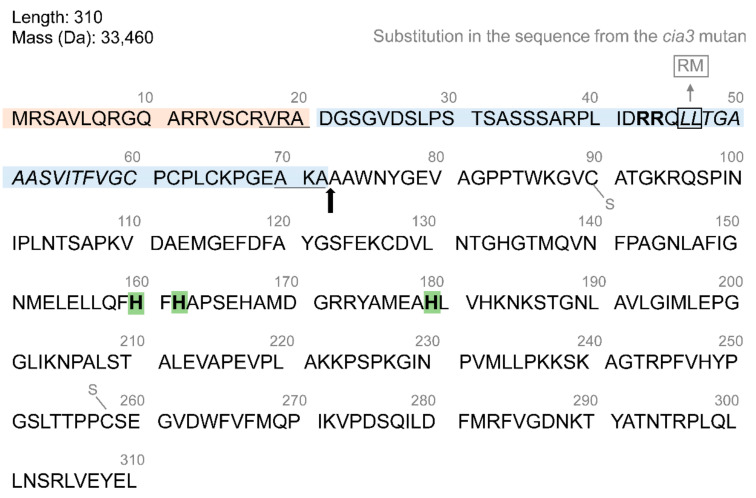
A full-length amino acid sequence of the CAH3 protein of *C. reinhardtii*. Two transport peptides are indicated by orange and blue backgrounds and three His of the active center are indicated by green backgrounds. The arrow shows the cleavage site between transport peptides and the mature protein. The cleavage sites for stromal and lumenal peptidases are underlined. The hydrophobic region is shown in italics. The twin Arg motif in the second transport peptide is shown in bold. Two Cys involved in the formation of a disulfide bond are indicated by S–. Two amino acids substituted in the second transport peptide of the *cia3* mutant are shown in the gray frame.

The amino acid sequence of the full-length chloroplast-targeted polypeptide of CA, named CAH3 (Figure 1), indicates a relation to the α-family with 40% identity to the N-termini of human CAII [20,21]. The sequence of the mature CAH3 protein showed 30–40% identity with α-CAs and up to 90% within the conserved domains, including three His residues functioning as ligands to a Zn atom. The predicted isoelectric point was ~7.78 [20]. The first 72 amino acids of the full-length sequence of the protein include two transport peptides or the stromal-targeting domain formed by 20 amino acids with a cleavage site, Val-Arg-Ala, for stromal peptidase and the lumen-targeting domain of 52 amino acids with a cleavage site, Ala-Lys-Ala, for lumen peptidase following the hydrophobic region [20].

A comparison of the sequences for the *Cah3* genes obtained from CC-503 and the *cia3* mutant *C. reinhardtii* revealed that two point mutations in the second transit peptides resulted in substitutions of two Leu in a pair into Arg and Met (Figure 1). Importantly, these are placed directly at the beginning of the hydrophobic region and almost immediately after the twin arginine motif, which is critical for thylakoid protein translocase in the ∆pH-driven pathway [38,39]. The same motifs in transit peptides were found in sequences of the PsbP, PsbQ, and PsbT subunits of PSII, as well as sequences of the PsaN subunit of PSI and other proteins of the lumen [38,39]. It has been shown that the amino acid substitution in the twin arginine could strongly reduce (by >100-fold) the translocation rate of the precursor protein or completely block it [38]. Interestingly, the presence of two point changes near the twin arginine motif in the case of CAH3 is enough to completely disrupt the transport of the precursor protein from the stroma to the thylakoid lumen. This explains the absence of the 30 kDa photoaffinity-labeled peptide in cell lysates from *cia3* [36], as well as the insignificant CA activity associated with the chloroplast, which is not suppressed by EZA [32,33]. 

The production of primary antibodies [20,40] made it possible to study the CAH3 content in cells and different preparations obtained from cells to identify the localization of the protein. The association of the CAH3 protein with thylakoid membranes was clearly shown [20,23,40]. Moreover, many signals from the antibodies were observed in membrane preparations enriched by PSII, which directly correlated with the values of CA activity, while photosystem I fragments did not contain CAH3 at all [22,23]. This localization of CA indicated the possible involvement of the CAH3 protein in the function of PSII [20,22,23], in spite of the fact that the participation of CAH3 in CCM was also proposed [20,22]. 

One of the questionable points of the works presented here was related to the detection of CAH3 in *cia3* cells, as well as thylakoids, and the PSII-enriched membranes isolated from this mutant with custom-produced primary antibodies [20,23]. Mitra et al. [40] even compared “Old” (used in [20,23]) and “New” antibodies, produced by their group, and showed the presence of an additional strong unspecific signal from the protein close to CAH3 in molecular weight when “Old” antibodies were used. The “New” antibody indeed did not detect CAH3 in *cia3* cells, in contrast to the wild-type (WT) 137c strain. In agreement, the commercially available primary antibody against CAH3 (Agrisera, Sweden) does not detect CAH3 in PSII preparations from the *cia3* mutant, while a significant amount of CAH3 is observed in PSII-enriched membranes from CC-503 (wall-deficient mutant) [10,25,26]. Based on the fact that the *Cah3* gene is constitutively expressed [14,16] and only slightly (or even not [14]) regulated by CO_2_ level, the absence of CAH3 in *cia3* cells indicates the fast proteolysis of the precursor CAH3 protein in chloroplast stroma. 

A study performed by Mitra et al. [40] of the immunolocalization of CAH3 with transmission electron microscopy using the “New” antibodies and protein A conjugated to colloidal gold (the secondary antibodies) showed a high distribution of CAH3 throughout the thylakoids of the chloroplast in the cells of the WT 137c strain. In addition, the signal from gold particles related to CAH3 proteins was clearly detected in association with thylakoids penetrating the pyrenoid matrix (tubules). The cells of the *cia3* and *ca-1* (has a stop codon in the *Cah3* gene) mutants contained extremely low counts of gold particles, in agreement with Western blot results for CAH3 [40]. 

In contrast, other groups using the “Old” antibodies showed a much higher concentration of gold particles inside the pyrenoid of WT 137c cells in association with tubules [24,41]. The gold particles were also observed in the pyrenoid area of *cia3* cells without a clear association with tubules, which was in agreement with the Western blot-detected CAH3 protein in *cia3* cells [41]. Taking into account the above-mentioned possible unspecific detection of a protein close to CAH3 in molecular weight using the “Old” antibodies [40], the obtained results need to be clarified. The “Old” antibodies were also used in the work, which showed a relocation of CAH3 proteins (identified using transmission electron microscopy) phosphorylated at a low CO_2_ level from the stroma thylakoid to the tubules of the pyrenoid. The authors of [37] noted that the maximum amount of CAH3 (gold particles) in the pyrenoid could reach ~37% at low CO_2_, but at high CO_2_ the value was only ~19%. At the same time, Western-blot showed a similar content of CAH3 in fractions of extrinsic proteins obtained from thylakoids of cells grown at high CO_2_ and adapted to low CO_2_. Using transmission electron microscopy, Mitchell et al. [42] showed a light-induced increase in CAH3 (gold particles) content in the pyrenoid from ~22% up to ~40% (by ~45%) with the use of a commercially available primary antibody. Interestingly, the pyrenoid area was also increased by ~37% in such cells (from 1.4 µm^2^ to 2.2 µm^2^). 

In spite of the early obtained data about the constitutive expression of the *Cah3* gene [14,16], which was independent of a low CO_2_ level as well as CIA5 regulation (the same was shown for the *Cah6* gene), Tirumani et al. [43,44] showed an almost complete decrease in *Cah3* transcripts upon adaptation of cells to light or high CO_2_ in a synchronized culture grown in a 12 h light/12 h dark regime. The CAH3 protein level was also changed, with the maximum observed at 6–12 h in the light and the minimum at 9–12 h in the dark [43]. The use of the immuno-fluorescence approach and antibodies against CAH3 indicated a diffuse pattern of the protein distribution among the thylakoids in the dark, with the maximum concentration of CAH3 in the pyrenoid area in the light, which partially correlated with data obtained by Mitchell et al. [42]. However, in another work, Mitchell et al. [45] showed no significant differences in *Cah3* gene expression levels under cell (WT 2137) adaptation to low CO_2_ in agreement with previously obtained data [14,16]. In addition, the in vivo detection of the signal from constructs encoding the CAH3 protein fused to a fluorescent tag showed a uniform distribution of CAH3 throughout the chloroplast (thylakoids), including the pyrenoid area in *C. reinhardtii* cells (CC-4533) grown under light and adapted to an ambient CO_2_ level for 24 h [19,46].

## 4. Production of the Recombinant Protein and Its Crystal Structure

There have been only two attempts to produce a recombinant protein of CAH3 (rCAH3) to date [25,40]. However, the same 349-amino-acid-long pET32-Xa-CAH3 plasmid was used in both studies. The construction contained a mature part of CAH3 and a 156-amino-acid N-terminal extension including His_6_-tag thioredoxin and a Factor Xa cleavage site (trx-His_6_-Xa-CAH3 fusion protein). Purified rCAH3 showed a high CA activity comparable with that of native CAH3, CAH1, and rCAH6, while it was significantly higher than the activity observed for rCAH7 and rCAH8 (Table 2). Interestingly, there were no differences in CA activity between the Factor X-cleaved rCAH3 protein and the uncleaved fusion protein [40]. 

The sensitivity of rCAH3 to sulfonamide inhibitors was extremely high with IC_50_ in nM concentrations, which was similar to that of native CAH3 (Table 2). Close values of IC_50_ were obtained for two other α CAs of *C. reinhardtii*, CAH1 and CAH2. The monovalent anions cyanide and azide suppressed the activity of rCAH3 in the μM range. The activity of rCAH3 was sensitive to sulfhydryl-reducing agents; thus, the incubation of rCAH3 with 10 mM of Cys, β-mercaptoethanol, or dithiothreitol decreased CA activity by ~47%, ~60%, and ~80%, respectively [40]. The same was observed for CAH1 and CAH2 (Table 2) [29,47]. However, 2 mM of dithiothreitol led to a complete inhibition of rCAH3 in [48]. The suppression of the CA activity of rCAH3 using dithiothreitol was completely reversible upon the oxidation of the SH groups of Cys90 and Cys258 (Figure 1), although the process required much longer times and a full restoration of CA activity was observed after 4 h [48]. The incubation of the rCAH3 protein for 15 min at different temperatures showed the optimum to be around 32–33 °C and a loss of a half of the activity above 43 °C [40].

The use of membrane inlet mass spectrometry (MIMS) based on monitoring the ^18^O exchange rate between C^18^O_2_ isotopes (dissolved in added air-saturated H_2_^18^O) and water allowed the pH dependence of rCAH3 CA activity to be investigated [48], while most of the commonly used methods based on time-resolved changes in pH are not suitable for this. The obtained results indicated that rCAH3 had an optimum CA activity near pH 6.5, with a residual CA activity of ~60% at pH 7.0 and of ~80% and ~45% at pH 6.0 and pH 5.5, respectively. At the same time, bovine CAII showed maximal CA activity at pH 7.0 with small residual CA activity at pH below 6.0. 

**Figure 2:**
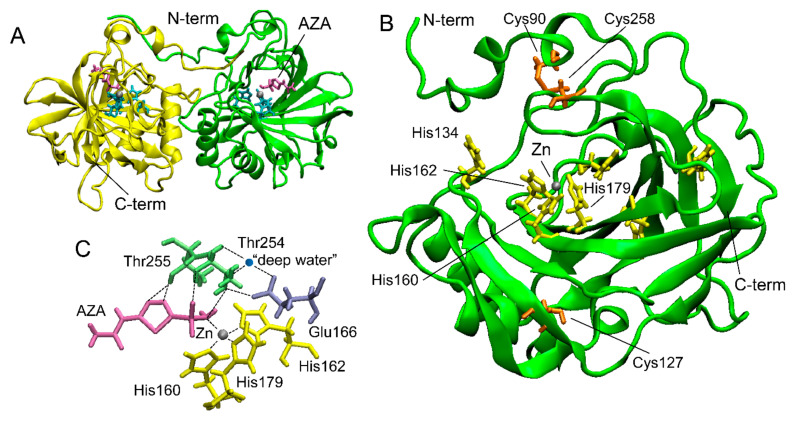
The crystal structure of rCAH3 obtained at pH 4.1 in a complex with AZA (PDB ID: 4xiw, https://www.wwpdb.org/pdb?id=pdb_00004xiw (accessed on 22 November 2023)). (**A**) A typical dimer of two rCAH3 molecules formed under crystallization. Four such dimers form each unit cell [48]. (**B**) The structure of an rCAH3 monomer. (**C**) Relative positions of amino acid residues, Zn, “deep water”, and AZA in the active center of rCAH3. His are shown in cyan (in **A**) or in yellow. Cys are shown in orange. Thr and Glu are shown in green and in violet, respectively. Zn and water are represented by gray and blue spheres, respectively. A molecule of AZA is shown in magenta. The positions of N- and C-terms are indicated.

Two crystal structures of rCAH3 in complex with AZA and phosphate ions were determined with 2.6 Å and 2.7 Å, respectively [48]. In both cases, each unit cell contained four dimers of rCAH3 (Figure 2A). Such packing is unusual for α-CAs. In accordance with known structures of other α-CAs, a monomer of rCAH3 is formed around a central core by 10-stranded β-sheets with two α-helices near one side of the sheet (Figure 2B). The full length of the molecule is ~45 Å. The active site is represented by an 8–9 Å-deep hydrophobic cavity where the catalytic Zn ion is coordinated by the residues of three His (160, 162, 179). The water molecule (“deep water”) structurally conserved among α-CAs has hydrogen bonds with Thr254, Glu166, and Tyr77 (Figure 2C). The monomer of rCAH3 has three Cys (90, 127, 258) with the formation of a disulfide bond between Cys90 and Cys258. As mentioned above, this disulfide bond is important for the CA activity of the enzyme.

**Table 2:** Maximal CA activities (WAU mg^−1^) and influences of inhibitors and reductants of the disulfide bond obtained for some recombinant (r) and native (n) proteins of *C. reinhardtii* as well as bovine CAII. IC_50_—half-maximal inhibitory concentration (M); DDT—dithiothreitol; ME—β-mercaptoethanol; ↓ or ↑—decrease or increase in CA activity; α and β indicate the CA family.

Protein	WAU	IC_50_	DTT/β-ME	Ref.
rCAH3 (α)	750 (1600)	AZA, 8 × 10^−9^ EZA, 6 × 10^−9^ Azide, 3.2 × 10^−5^ Cyanide, 5.9 × 10^−5^	↓	[25] (Shutova et al., 2008, unpublished data)
rCAH6 (β)	940	EZA, 2 × 10^−6^ Azide, 1.5 × 10^−5^ Cyanide, 5 × 10^−5^	no	[25]
rCAH7 (β)	3.1	AZA, 4.4 × 10^−5^ EZA, 4.4 × 10^−4^ Azide, 3.2 × 10^−5^ Cyanide, 6.1 × 10^−4^	↑	[16]
rCAH8 (β)	4.2	AZA, 1.7 × 10^−7^ EZA, 4.1 × 10^−4^ Azide, 1.9 × 10^−3^ Cyanide, 1.5 × 10^−5^	no	[16]
rLCIC, rLCIB, rLCIC-LCIB (β)	no	–	–	[13]
nCAH3 (α)	2040	AZA, 12 × 10^−9^	–	[20]
nCAH1 (α)	2580	AZA, 3 × 10^−9^ EZA, 2 × 10^−9^	↓	[29,47,49]
nCAH2 (α)	3300	AZA, 3 × 10^−9^ EZA, 2 × 10^−9^	↓	[29]
r-Bovine CAII (α)	9333	AZA, 1.4 × 10^−8^ EZA, 1.2 × 10^−9^ Azide, 1.1 × 10^−3^ Cyanide, 4.9 × 10^−5^	no	[40,50]

In addition to the three His of the active center, the monomer of rCAH3 has His134 on the bottom edge of the cavity (Figure 2). As is known, a similar His (His64) plays a role as a proton shuttle in human CAII between a bulk solution and a Zn-bound solvent molecule with a rate of more than 10^5^ s^−1^ [51]. The same His64 is also present in bovine CAII (https://www.wwpdb.org/pdb?id=pdb_00001v9e (accessed on 22 November 2023)). A structural analysis of human CAII at different pH values showed no major pH-induced conformational changes in the active site [52], while His64 could be found in the “out” (at pH 5.7) or “in” (at pH 8.5) conformation oriented away or toward the Zn ion, respectively [51,52]. Whether the unusual pH optimum of rCAH3 is caused by His134 is not yet known. In addition, rCAH3 has no His at the N-term (Figure 2), in contrast to human and bovine CAIIs that have 5 His among the first 20 amino acids of the N-term oriented to the cavity. The monomer of rCAH3 does not contain the three loop regions present in human and bovine CAIIs, which can explain its shallow and broader cavity with a more hydrophobic entrance as compared to human and bovine CAIIs, which are probably needed for the interaction of the protein with the thylakoid membrane [48].

## 5. Involvement CAH3 in CCM

**Figure 3:**
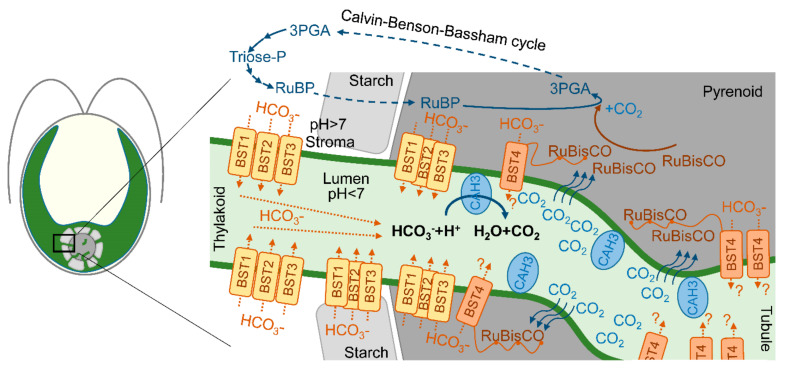
A scheme of proposed localization of CAH3 in the lumen of intrapyrenoid thylakoids (tubules) and a possible role of CAH3 in CO_2_ production, which passes the thylakoid membrane to the pyrenoid matrix where it can be captured by RuBisCO in the Calvin–Benson–Bassham cycle. A simplified Calvin–Benson–Bassham cycle is adapted from [56]; RuBP—ribulose-1,5-biphosphate; 3PGA—3-phosphoglyceric acid. Putative localization and roles of four bestrophin-like proteins (BST1-3 and BST4 (RuBisCO binding membrane protein)) as HCO_3_^−^ transporters of the thylakoid membrane are adapted from [53,57].

It was clearly shown that the *cia3* (CAH3-deficient) mutant requires a high CO_2_ level for its growth [20,53,54]. Thus, the direct or indirect participation of CAH3 in the CCM of *C. reinhardtii* is often proposed. At the same time, the authors of [20,22] suggested that PSII can drive the lumenal part of CCM via the CA activity of PSII-associated CAH3, providing CO_2_ for carboxylation byRuBisCO. However, using transmission electron microscopy, Mitra et al. [40] showed the presence of CAH3 not only in association with stromal thylakoids but with thylakoids (tubules) penetrating the pyrenoid where PSII complexes are exactly absent. Thus, CAH3 can be involved in CCM independently of PSII as single molecules. The proposed role of CAH3 was in the acceleration of the HCO_3_^−^ dehydration reaction (HCO_3_^−^ + H_2_O → H^+^ + CO_2_) in the lumen of tubules located inside the pyrenoid matrix. Produced CO_2_ easily passes through the thylakoid membrane and can be used by RuBisCO (Figure 3) [24,40,55]. The presence of CAH3 in the pyrenoid area was also shown by other groups using transmission electron microscopy [24,37,41,42], as discussed above (Section 3).

The in vivo monitoring of ^16^O_2_, ^18^O_2_, and CO_2_ fluxes using MIMS indicated an absence of limitations in the electron transport in PSII of the *cia3* mutant under low-CO_2_ conditions compared to WT (CC-400) [58], while net CO_2_ uptake was greater in the mutant [58], which was a result of the known higher Ci accumulation inside cells without CAH3 (Table 3) [34,55]. Nevertheless, if WT cell was able to consume all Ci of the solution, the *cia3* mutant cells could only draw down up to ~35 μM of Ci [58]. 

RuBisCO in the *cia3* mutant showed extra sensitivity to O_2_, with a higher CO_2_ compensation point that was strongly influenced by the O_2_ concentration. A study of metabolites indicated a much higher ribulose-1,5-biphosphate (RuBP) pool size in the *cia3* mutant at 200–600 μM of Ci, while the pool size of 3-phosphoglyceric acid (3PGA) showed an inverse pattern [58]. In *C. reinhardtii*, both of the intermediates are mostly localized in the pyrenoid matrix (when the pyrenoid is formed), where PuBP is converted to 3PGA by RuBusCO with CO_2_ assimilation during the Calvin–Benson–Bassham cycle (Figure 3). Thus, the absence of CAH3 in the lumen of tubules can suppress the supply of CO_2_ for fixation by RuBisCO impairing the CCM. It should be noted that the authors of [58] used relatively distinct strains (CC-400, *cia3*) compared, for example, with the works by [10,23,25,26,27], where closely related strains (CC-503, *cia3*) obtained from WT CC-137c [59] were used. Cells were not preadapted to low-CO_2_ conditions, and measurements required a long assay period to complete each data set.

**Table 3:** Intracellular Ci accumulation after 80 s (* 60 s) by WT and different CCM mutants of *C. reinhardtii* acclimated to low CO_2_. Mutants *ca*-1-12-1C, WT-*su6*, and *ad1-su6* are deficient (def.) in terms of CAH3 production (underlined).

Strains	Initial External Ci, μM	Internal Ci, mM	Ref.
WT 2137	80	~1.0	
+50 µM EZA	80	~10.0	[34,35]
*ca*-1-12-1C	80	~13.2	
Strain 90	20	~0.25 *	
+50 µM EZA	20	~1.10 *	[60]
+50 µM AZA	20	~0.25 *	
WT CC-125	50	1.80 ± 0.33	
*ad1* (LCIB–def.)	50	0.26 ± 0.08	[55]
WT-*su6*	50	7.60 ± 1.25	
*ad1*-su6	50	6.15 ± 1.15	
*cia5*	50	0.19 ± 0.05	
WT CC-125	50	~1.25	
*ad1* (LCIB–def.)	50	~0.20	[61]
WT D66	50	~0.95	
*bsti-1* (BST1–def.)	50	~0.33	[53]
*bsti-2* (BST2–def.)	50	~0.50	
*bsti-3* (BST3–def.)	50	~0.51	
WT D66	25	~0.38	
*cah4*/*5-1* (CAH4/5–def.)	25	~0.10	[54]
*cah4*/*5-2* (CAH4/5–def.)	25	~0.20	

The LCIB (together with LCIC) protein of the chloroplast stroma is known as a participant of the CCM in *C. reinhardtii* [13]. A study with an LCIB-deficient mutant *ad1* clearly shows an “air dier” phenotype when cells are unable to grow under low CO_2_ [55,61]. To reach the maximum photosynthetic O_2_ evolution, the *ad1* mutant acclimated to low CO_2_ required an addition of more than 400 μM NaHCO_3_ (~100 μM for WT), but even in this case the obtained value was lower by ~40% compared to the WT [55,61]. In agreement, the intracellular accumulation of Ci by the *ad1* mutant is ~6 times lower compared to that of the WT (Table 3). Two transformants of the *ad1* mutant, *ad-su6* and *ad-su7*, with deletions in the *Cah3* gene that stop its expression, were surprisingly able to grow under low CO_2_ [55]. They accumulated 3.5–4 times more intracellular Ci compared to that of the WT or 24–29 times more compared to that of the *ad1* mutant, which results were consistent with that observed for the CAH3-deficient mutant *ca*-1-12-1C (or with experiments with the addition of EZA, which is able to penetrate through bilipid membranes and inhibit CAH3 in contrast to AZA) (Table 3). Such a high level of Ci may be needed to sufficiently provide CO_2_ to RuBisCO in the absence (or inhibition) of CAH3, as mentioned above; however, this suggestion requires further investigation. The authors of [55] noted that CAH3 mutations are epistatic to LCIB mutations and that LCIB activity should be downstream of CAH3. 

One of the suggested problems associated with the involvement of CAH3 in CCM in tubules is the need to supply a lot of HCO_3_^−^ ions for the dehydration activity of CA. While most of the Ci in the stroma are represented as HCO_3_^−^ ions due to the alkaline pH, these are not able to pass the thylakoid membrane, unlike CO_2_ molecules. In addition, the acidic pH of the lumen favors the conversion of Ci into CO_2_ [10]. Recently, three similar bestrophin-like (BST1-3) proteins of the thylakoid membrane were suggested as HCO_3_^−^ transporters [53]. The visualization of BST1-3 localization via protein-linking Venus fluorescence showed a distribution of the signal among the thylakoids of the chloroplast concentrated around the pyrenoid. Moreover, the presence of BST1-3 extended into the tubules. This localization of transporters can explain the supply of HCO_3_^−^ for CAH3 in the tubules for CCM operation (Figure 3). Low CO_2_ induced an increase in the transcript levels of all three genes encoding BST1-3. At the same time, the knockout mutants (*bsti-1*, *-2*, *-3*) accumulated 47–63% less intracellular Ci compared to the WT (D66) (Table 3), while they were also able to grow at a low CO_2_ in contrast to the *cia3* mutant. Another BST4 protein (also known as RuBisCO-binding membrane protein) exclusively localizes in the tubules of the pyrenoid (Figure 3). BST4 has a conserved bestrophin domain similar to that observed for BST1-3; therefore, its role in HCO_3_^−^ ions for CAH3 activity in CCM was proposed [57]. However, the data obtained so far show that BST4 is not a component of CCM, but it may be involved in the pH regulation of the lumen in addition to the main function of BST4 of binding RuBisCO to tubules [62].

Interestingly, the knockout of two mitochondrial Cas, CAH4 and CAH5, which are known as CCM participants, also led to a decrease in the accumulation of intracellular Ci by up to 74% as compared to the WT (D66) (Table 3), while the mutants *cah4*/*5-1* and *cah4*/*5-1* were able to grow at low CO_2_ [54]. Thus, CAH3 mutation leads to an overaccumulation of intracellular Ci in contrast to mutations of other CCM participants, but the molecular mechanism is still unknown. The inability of the CAH3-deficient mutants *cia3* and *ca*-1-12-1C to grow at low CO_2_ [20,53,54] makes this question more complicated. 

The role of CAH3 in CCM was also studied using mutants with a blocked formation of the pyrenoid (*pyr−*). As shown in [45], the absence of the pyrenoid did not influence the expression level of the *Cah3* gene. The same was observed even after the cells were adapted to low CO_2_. Mass spectrometry analysis showed that many proteins related to CCM were less abundant in *pyr−* cells adapted to low CO_2_ compared to *pyr+* cells, while the difference observed for CAH3 content was not statistically significant. In spite of the fact that *pyr−* mutants are limited in terms of the enzymatic activity of RuBisCO at low CO_2_, starch granules, as well as a complex of modified thylakoids (knitted tubules), can be observed in the usual area of the pyrenoid formation [63]. It is likely that part of the CAH3 molecules can be localized in those tubules with residual CCM function.

## 6. Functional and Structural Interconnection between CAH3 and PSII 

**Figure 4:**
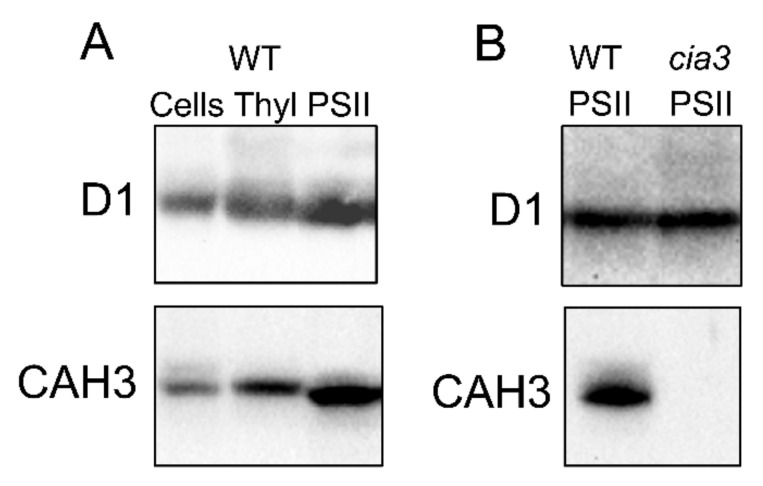
Results of Western blot analysis with use of primary antibodies against D1 and CAH3 proteins of (**A**) whole cells, thylakoids (Thyl), and PSII-enriched membranes (PSII) isolated from WT (CC-503), as well as of (**B**) PSII-enriched membranes (PSII) isolated from WT (CC-503) and a *cia3* mutant. Samples were loaded at 1.5 μg Chl per lane. For a complete description of the analysis, see [10,26].

The role of CAH3 CA activity in supporting of the optimal PSII photochemistry was proposed by Karlsson et al. [20] together with the first description of the novel α-CA (CAH3) of the thylakoid membrane. Later, the results of Western blot analyses performed in many studies clearly showed a high abundance of CAH3 in PSII-enriched preparations (Figure 4A; Section 3), indicating an interconnection between the activities of CAH3 and PSII. 

The use of artificial acceptors, such as 2,6-dichloro-1,4-benzoquinone (DCBQ) paired with potassium ferricyanide (FeCy), which are capable of directly taking electrons from the PSII acceptor side even in the case of whole cells [64], allows for PSII function to be studied independently of other participants of the electron transport chain, as well as independently of the carboxylation status of RuBisCO. This approach clearly demonstrates the dependence of the stability of PSII function, and especially of WOC activity, on the presence of CAH3. It was shown that PSII in thylakoids from the *cia3* mutant had much stronger photoinhibition (at 600 μmol photons m^−1^ s^−1^, 10 min) compared to that from WT (CC-503), and the presence of EZA during illumination decreased the functional stability of PSII from WT to the level of *cia3* [23]. On the contrary, the addition of an excess of HCO_3_^−^, which may imitate dehydratase CAH3 activity (HCO_3_^−^ + H_2_O → H^+^ + CO_2_), protected the PSII of *cia3* from photoinhibition. The same stronger suppression of PSII in the *cia3* mutant compared to the WT (CC-400) by the light of >400 μmol photons m^−1^ s^−1^ was observed in whole cells using MIMS, while the authors of [58] concluded that the presence of RuBisCO inhibition caused by O_2_ accumulated in a closed chamber. Nevertheless, the study of chloroplast morphology in vivo under continuous illumination using moderate-intensity light (200 μmol photons m^−1^ s^−1^) indicated the development of an unusual morphology in the case of the *cia3* mutant in contrast to the WT [65]. The clear separation of the chloroplast into lobes and the basal region was diminished with the formation of a net-like structure inside the cell. The main Chl fluorescence intensity was shifted from the edges, demonstrating a relocation of a major part of PSII. Since the WT and the *cia3* mutant cells had the same photosynthetic activity of PSII, as well as the same content of D1 protein, the author of [65] proposed an initiation of a peculiar protection mechanism in *cia3* cells against the photoinhibition of PSII. This consists of an absorption of excess light by PSII remaining near the edges of the chloroplast to provide optimal light conditions for the main part of PSII located under them. Thus, PSII of *cia3* mutant cells is indeed more sensitive to light. 

The study of PSII-enriched membranes isolated from WT (CC-503) and *cia3* mutant cells grown under optimal conditions including a supply of 5% CO_2_ surprisingly showed no significant differences in their main structural and functional futures [26]. The preparations contained a similar amount of PSII per the same amount of Chl, as can be seen from the Western blot analysis against D1 (PsbA) (Figure 4B) and PsbO proteins [10,25,26], as well as from the cytochrome b559 and Mn atom contents [10]. This is in spite of the fact that PSII from the *cia3* mutant did not contain any CAH3 (Figure 4B). The curves of the pH dependence of O_2_-evolving activity showed the same shape below 6.5. However, differences can be seen at pH above 6.5, where the WOC activity in PSII from the *cia3* mutant was more suppressed compared to preparations from WT cells (maximum difference ~20% observed at pH 7.0) [10]. The addition of AZA or EZA decreased the photosynthetic activity of PSII from WT cells to that detected for PSII from the *cia3* mutant, indicating the role of the CA activity of CAH3 in this effect. On the other hand, the addition of 0.5 мM HCO_3_^−^ completely restored the O_2_-evolving activity of PSII from the *cia3* mutant up to the level of PSII from WT cells, which shows the importance of catalyzing the HCO_3_^−^ dehydration reaction in agreement with that mentioned above. The exact influence of the CA activity of CAH3 on the function of the WOC was clearly shown in the study with 2,6-dichlorophenolindophenol. The rate of its photoreduction by PSII from H_2_O (the WOC function) at pH 7.0 was significantly lower in PSII from the *cia3* mutant compared to PSII from WT, while the presence of 1,5-diphenylcarbazide, which is able to effectively donate electrons to PSII separately from the WOC, completely removed this difference. In addition, the CA activity of CAH3 also protected the function of the WOC from irreversible inactivation under pH higher than 7.0 [10].

The depletion of CO_2_/HCO_3_^−^ from the medium (Ci-free) allowed a similar effect at pH 5.5 to be identified [25]. The O_2_-evolving activity of PSII from WT under these conditions could be increased by ~12% through the addition of 2 mM HCO_3_^−^ and by ~36% in the case of PSII from the *cia3* mutant, i.e., the difference reached ~24%. The measurement of flash-induced O_2_ evolution patterns showed a decrease in the turnover efficiency of the WOC in PSII from both the WT and *cia3* mutant in the Ci-free medium. The increase in the miss parameter was higher in PSII from the *cia3* mutant (~7%) compared to PSII from the WT (~4%), and the difference should be obviously higher under continuous illumination and in vivo [48]. The reconstruction of PSII from the *cia3* mutant by the addition of rCAH3 in Ci-free medium resulted in a much stronger stimulation (~70%) of O_2_ evolution when HCO_3_^−^ was added [25]. Moreover, this high activity was stable even under continuous illumination (at 200 μmol photons m^−1^ s^−1^, 180 s) of the samples. The dependence of the O_2_-evolving activity of PSII on rCAH3 content showed the maximal value at a molecular stoichiometric ratio of 1:1. The results of the Western blot analysis indicated a complete binding of rCAH3 molecules with the membrane fraction of PSII-enriched membranes [25]. 

**Figure 5:**
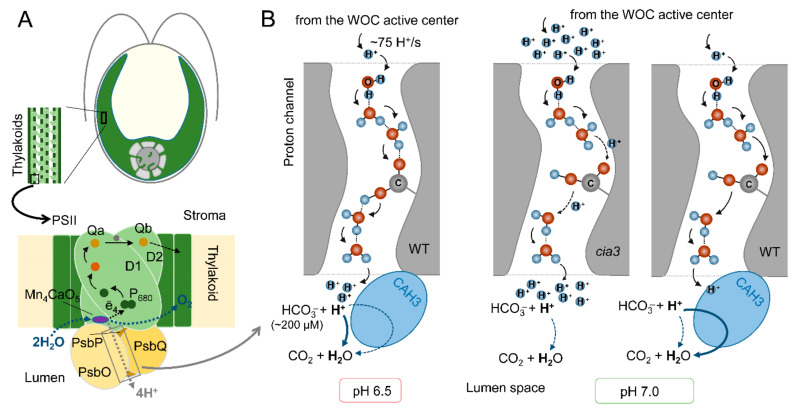
The proposed role of CAH3 activity in the acceleration of proton removal from the active center of the WOC of PSII from the WT and *cia3* mutant at optimal pH 6.5 and nonoptimal pH 7.0: (**A**) A schematic representation of the PSII complex including a dimer of D1 and D2 proteins with cofactors of the electron transfer chain, proteins of the WOC (PsbO/P/Q), and Mn_4_CaO_5_ cluster, which is the active center of the WOC (for more information, see [66]). CAH3 protein is not indicated because its exact location near the WOC has not yet been established. (**B**) A possible involvement of CAH3 in the H^+^ “neutralization” near outlets of the proton channels in the lumen. A proton channel scheme was created based on the data from [67], where H^+^, water molecules, and an amino acid residue are presented. The thickness of the arrows indicates the effectiveness of proton transfer or of the dehydration reaction. For details, see the main text.

The inhibition of CAH3 activity and/or addition of HCO_3_^−^ to PSII-enriched membranes, imitating dehydratase activity, had no influence on WOC activity at pH 6.5, which is optimal for the operation of the WOC. Moreover, there were no differences in the rates of 2,6-dichlorophenolindophenol photoreduction by PSII isolated from both the WT and *cia3* mutant, nor was there an influence of 1,5-diphenylcarbazide [10]. All of these results indicated that PSII from the *cia3* mutant functioned at its maximum possible values, equal to those of PSII from WT, and that CAH3 CA activity had no influence on the function of PSII from the WT. Therefore, the functional interconnection between PSII and CAH3 becomes more pronounced at nonoptimal conditions for the WOC. In different studies [10,25], the same role of CAH3 for PSII was hypothesized, which is the acceleration of proton removal from the active center of the WOC to the thylakoid lumen (Figure 5). 

Photosynthetic water oxidation results in the production of 4H^+^ per O_2_ (2H_2_O → 4ē + O_2_ + 4H^+^) (Figure 5A). A calculation based on the O_2_ evolution activity of PSII preparations (280 μmol O_2_ (mg Chl h)^−1^) indicated a H^+^ production rate of ~75 H^+^/s per PSII [10]. This requires a rapid removal of H^+^ from the active center of the WOC (to maintain its optimal activity) to the lumen via the proton channels including Cl^−^ atoms, water molecules, and amino acid residues of WOC proteins [67]. At an optimal pH for the WOC of 6.5, the effectiveness of H^+^ transfer via the channels is maximal and fully covers the proton production (Figure 5B). In addition, the Ci content in the medium at this pH was estimated as ~380 μM, with part of HCO_3_^−^ equal to 200 μM (~53%) [10], which is involved in a spontaneous dehydration reaction near outlets of the proton channels even in the absence of CAH3. An increase (or decrease) in pH induces conformational perturbations in proteins of PSII including the WOC [66], which is followed by changes in the spatial position of amino acids. This can result in increasing the distances between water molecules and amino acid residues involved in the formation of the proton channel and consequently in the suppression of H^+^ transfer (Figure 5B). At pH 7.0, the medium contains ~460 μM Ci, and the spontaneous dehydration reaction is unfavorable (~380 μM HCO_3_^−^, (~83%)) [10]. In the absence (*cia3*) or under the inhibition of CAH3, this leads to an accumulation of H^+^ near the outlets of the proton channels, which can be an additional suppressive factor for effective H^+^ transfer from the active center of the WOC, where local acidification inhibits its function. As shown, only a three- to fourfold increase in HCO_3_^−^ content can stimulate the photosynthetic activity of PSII, while the presence of active CAH3 (WT) accelerates the interconnection of H^+^ with HCO_3_^−^ of the lumen, supporting a high proton gradient in the channel, rapid H^+^ removal, and, thus, the optimal operation of the active center of the WOC. At pH 5.5, in addition to the consequences of conformational changes in PSII, the content of Ci is lower (~250 μM) with a very small part of HCO_3_^−^ (~40 μM, ~16%) [10]. However, a favorable spontaneous dehydration reaction (more favorable than that at pH 6.5, Figure 5B), even in the presence of only ~40 μM HCO_3_^−^ in the medium [10], was able to support a high level of H^+^ removal from the active center of the WOC through their “neutralization” near the outlets of the proton channels. The inhibition of WOC activity due to the suppression of H^+^ transfer to the lumen was observed under the depletion of CO_2_/HCO_3_^−^ from the medium [25].

In addition to the clear influence of the CA activity of CAH3 on PSII function and its stability, evidence was obtained about the presence of some conformational changes in PSII complexes assembled in the absence of the CAH3 protein, for example, in the case of the *cia3* mutant. The shape of the fast Chl fluorescence rise kinetic (OJ(I)P) measured for PSII-enriched membranes isolated from the *cia3* mutant had a more straightened J–P phase compared to the PSII from WT, resulting in a ~33% reduction in the Area parameter [26]. This indicated the suppression in the electron transfer between Qa and Qb on the PSII acceptor side (Figure 5A). It was proposed that this could be a result of some conformational changes in the native structure of the WOC induced by the loss of the CAH3 protein and transmission from the donor to the acceptor sides of PSII through the subunits of cytochrome b559 connecting with PsbP. (Other ways have been reviewed recently [66].) This is supported by the increased portion of the low-potential form of the cytochrome b559 in PSII from the *cia3* mutant [26]. Later, it was shown that the WOC in PSII from the *cia3* mutant is indeed characterized by lower functional stability under increased Cl^−^ content with a simultaneously stronger binding of the PsbP protein to the WOC [27]. Interestingly, the inhibition of CA activity had no influence on the obtained results, directly indicating the structural role of the CAH3 protein in the PSII complex of *C. reinhardtii*. This structural role of an active CA protein in a multiprotein complex has not been identified previously.

## 7. Incorporation of CAH3 into Higher Plants

The components of the CCM of algae are often considered to be a tool for improving the biomass production of higher plants due to chloroplasts saturation by Ci. The possibility of the CAH3 protein (or a ~37 kDa construct containing the catalytic part of CAH3 [68]) to localize in the thylakoids of higher plants [46,68] and increase CO_2_ assimilation was shown, which resulted in a larger size of plants and number of leaves and an acceleration of flowering [68]. The CAH3-containing mutants of tobacco plants showed an ~18% increase in both the maximal RuBisCO carboxylation rate and the maximal rate of linear electron transport, as well as higher levels of the effective quantum yield of PSII and the photochemical quenching of Chl fluorescence (qP) of PSII. The authors of [68] largely attributed this to a higher concentration of CO_2_ in the vicinity of RuBisCO. However, they also mentioned the possibility of CAH3 involvement in the acceleration of H^+^ removal from the active center of the WOC through catalyzing the dehydration reaction, as was proposed for *C. reinhardtii* PSII. On the one hand, the obtained results require further study to identify whether CAH3 is associated with PSII, which enhances linear electron transport resulting in an increase in the activity of RuBisCO, or, in contrast, whether it operates independently by removing the CO_2_ limitation on the carboxylation rate of RuBisCO. On the other hand, the results clearly show the possibility of using CAH3 as an effective tool for increasing the biomass production of higher plants, which can be used in agriculture in the future. 

## Figures and Tables

**Table 1:** CA activity (WAU mg Chl^−1^) of whole *C. reinhardtii* cells and isolated (solubilized) chloroplasts after growth at 5% (high) and ambient (low) levels of CO_2_. Note: 137c, wild-type strain; *cw15* and CC-503, wall-less mutants; *cia5*, strain with failed CCM, including periplasmic CAs; *cia3*, CAH3-deficient mutants (underlined); EZA, CA inhibitor ethoxyzolamide.

Strain	Whole Cells		Chloroplasts		Ref.
	High CO_2_	Low CO_2_	High CO_2_	Low CO_2_	
11/32b (protoplast)	224.5	1263.6	147.2	782.2	[30]
+150 µM EZA	57.3	303.3	57.3	280.8	
CC-503 cw92 (mt+)	20 ± 5	78 ± 10	5.3 ± 2.1	20 ± 2.1	[31]
137c (mt+)	–	740 ± 12.4	–	–	[32]
*cw15*	–	–	–	35.5 ± 2.6	
*cia5*/*cw15*	–	–	–	33.8 ± 1.9	
*cia3*/*cw15*	–	2.8 ± 7.8	–	2.8 ± 3.2	
*cw15*	48.3 ± 4.0	600 ± 50	18 ± 1.5	58 ± 4.5	[33]
+100 µM EZA	0.7 ± 0.06	0.5 ± 0.05	4.8 ± 0.3	3.9 ± 0.2	
*cia3*/*cia15*	45.5 ± 3.0	385 ± 30	5.0 ± 0.4	5.0 ± 0.4	
+100 µM EZA	0.5 ± 0.05	0.9 ± 0.04	3.3 ± 0.3	2.9 ± 0.2	
2137 mt+	89	616	–	–	[34,35]
*ca*-1-12-1C (=cia3)	98	103			

## Data Availability

The datasets generated during and/or analyzed during the current study are available from the corresponding author on reasonable request.
